# Spontaneous Expulsion of a Prolapsed Pedunculated Uterine Leiomyoma: A Rare Gynecologic Enigma

**DOI:** 10.7759/cureus.32832

**Published:** 2022-12-22

**Authors:** Twisha J Patel, Sandhya P Pajai, Kalyani S Mahajan

**Affiliations:** 1 Department of Obstetrics and Gynecology, Jawaharlal Nehru Medical College, Datta Meghe Institute of Medical Sciences, Wardha, IND

**Keywords:** benign smooth muscle tumor, pedunculated fibroids, leiomyomas, spontaneous expulsion, intramural fibroid

## Abstract

Uterine leiomyomas, also known as uterine fibroids, are smooth muscle tumors in the uterus, mostly benign in nature. They occur in the reproductive age group i.e. between 15 and 49 years. Asymptomatic in nature; rarely, they may be associated with symptoms like abnormal uterine bleeding, pelvic pain, and compression symptoms or secondary changes. Patients of the reproductive age group may be associated with infertility and recurrent pregnancy loss. Fibroids run in families and are associated with both estrogen and progesterone levels. Myomas produce symptoms depending on their site, size, position, number, or any secondary changes. The submucosal type of fibroid is associated with symptoms more commonly. Based on presenting symptoms, uterine leiomyoma can be managed medically or surgically. Here we present a case of a 32-year-old multigravida who had a spontaneous vaginal expulsion of a pedunculated intramural fibroid. Very rarely as in this case, complete expulsion of leiomyoma is seen. When it occurs in the reproductive age group, it may mimic many clinical conditions like incomplete or inevitable abortion. Such a case may also be associated with excess hemorrhage and can cause significant morbidity to the patient; hence it is essential to make an early diagnosis and necessary timely intervention.

## Introduction

Myoma is the most common uterine solid smooth muscle pelvic tumor with a multifactoral etiology [1]. Despite being monoclonal smooth muscle benign tumors, they are one of the major causes of morbidity in young females, thus being one of the major indications for surgical interventions as stated in a study of determining etiology, symptomatology, and diagnosis of uterine myomas [2]. It mostly occurs during the reproductive age group [3]. Tumor composition contains a large amount of extracellular matrix made of collagen, fibronectin, and proteoglycan. Most of the time uterine myomas are asymptomatic. They can be associated with symptoms like pelvic pain, irregular menses, and urinary problems. Although menorrhagia may or may not be associated with fibroids, other possibilities should always be ruled out. The fibroids can be classified into intramural, submucosal, subserosal, and cervical [1]. Submucosal myomas grow inside the myometrium and project toward the endometrial cavity. Sometimes these myomas are attached to a pedicle and so they can project out into the cervical canal and appear in the vagina.

## Case presentation

A 32-year-old woman with two live issues presented to casualty with complaints of excessive bleeding per vaginum associated with the passage of clots and abdominal pain which was severe in intensity, intermittent, and colicky in nature over a period of 12 hours. She also had a history of dysuria and foul smelly, itchy vaginal discharge. This patient had a 15-day history of continuous bleeding with three to four pads soaked/day along with the passage of clots and dysmenorrhoea. The patient had a history of menorrhagia in her previous cycles. She has operated on for tubal ligation seven years ago. On general physical examination, she was fairly built, fairly nourished, and appeared pale. Her temperature was 97°F, her pulse was 90 beats per minute, regular, normovolemic, and her blood pressure was 100/60 mm Hg. Cardiovascular and respiratory examinations were found to be within normal limits.

Upon examination of the abdomen, the uterus was palpable and firm. Speculum examination revealed a mass approximately 4 x 4 cm in the vagina, red in color with irregular borders and a smooth surface covered with clots and blood mixed with foul-smelling discharge. On vaginal examination a pedunculated mass was felt coming out of the internal os. Cervix was 3 cm dilated due to a thick peduncle. On sound test, the peduncle had origin in the uterine cavity and not cervical. Diagnosis of a pedunculated intramyometrial fibroid polyp with secondary infection was made clinically.

**Figure 1:**
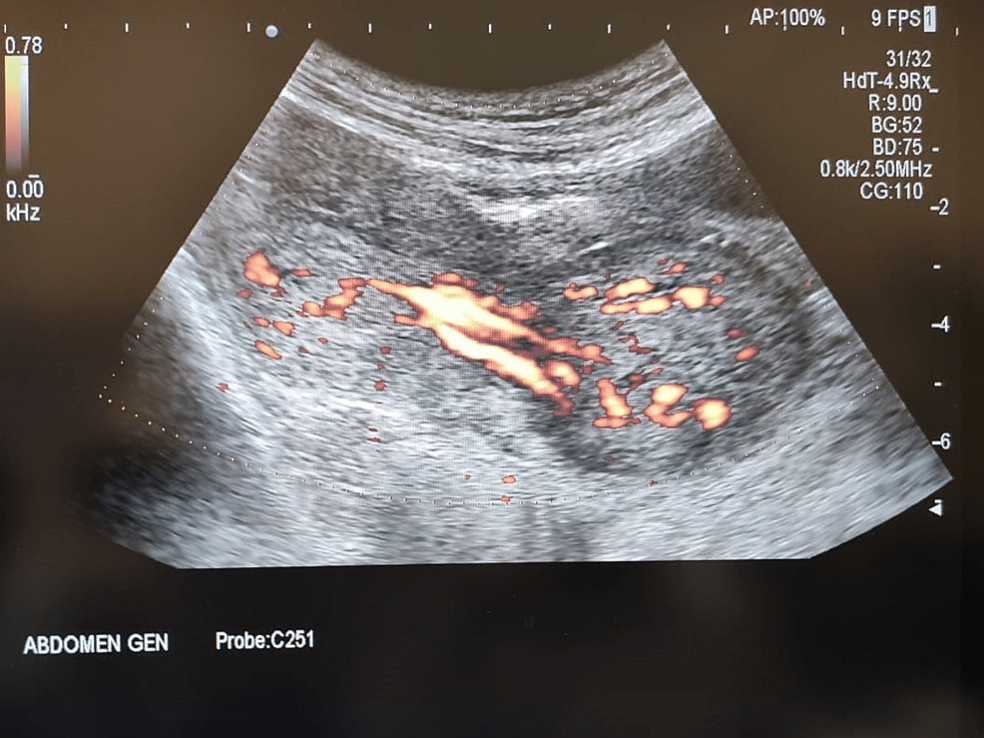
Bulky uterus with well-defined circumscribed heterogeneous hypoechoic lesion

**Figure 2:**
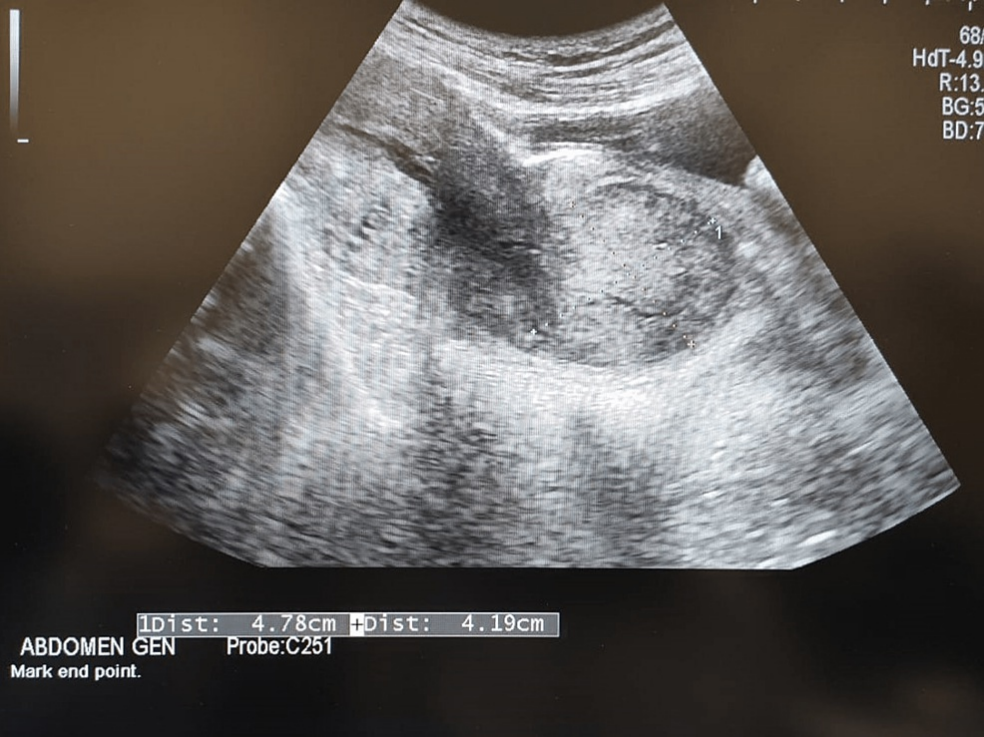
USG dimensions of fibroid USG: ultrasonography

**Figure 3:**
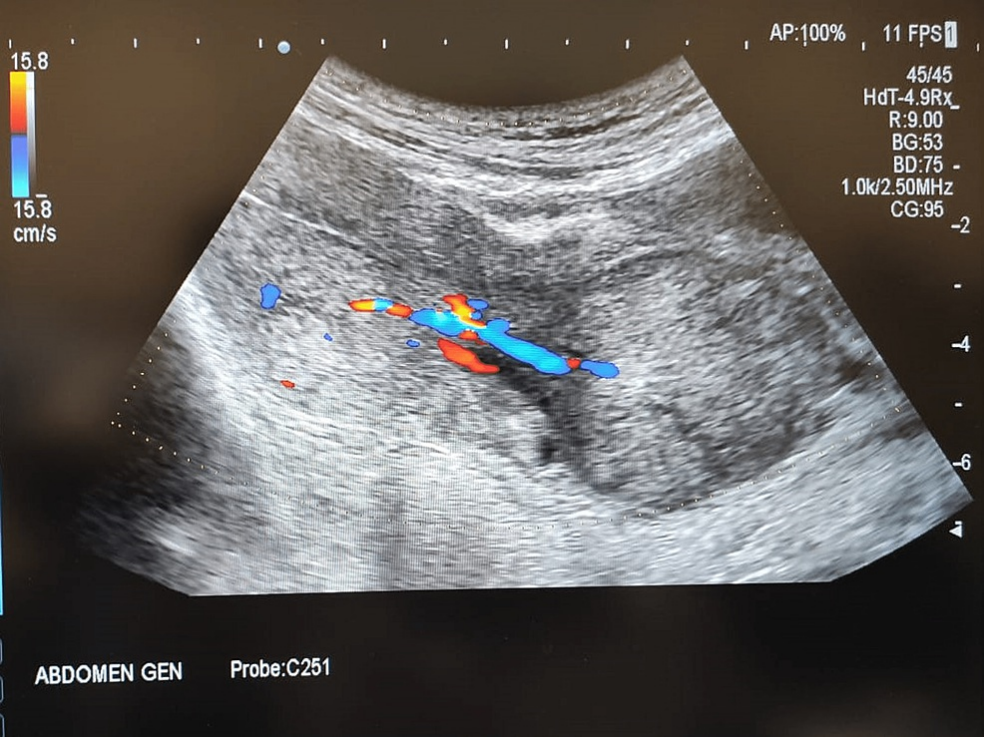
Vascular pedicle

The patient was admitted and all routine investigations were sent. She received ultrasonography, showing a bulky uterus 13.6 x 7 x 5.2 cm in size with evidence of a well-defined circumscribed heterogeneously hypoechoic lesion of size 33 x 22 mm in the fundal region (Figures 1, 2). There is evidence of a vascular pedicle noted extending from the mass (Figure 3). Impression likely of a fibroid polyp. Her labs were hemoglobin (Hb): 7.7 gm/dl; total leukocyte count (TLC): 18200/cumm; platelet (PLT): 5 lakh/cumm; random blood sugar (RBS): 96 mg. HIV and hepatitis B surface antigens (HBsAg) were non-reactive. Two unit-packed red cells were arranged for transfusion. Higher antibiotic coverage was given. Analgesics and tranexamic acid were administered. While the pre-operative preparations were being done, the patient had severe pain in the lower abdomen and a sensation of bearing down with the passage of clots and bleeding per vaginal (PV).

**Figure 4:**
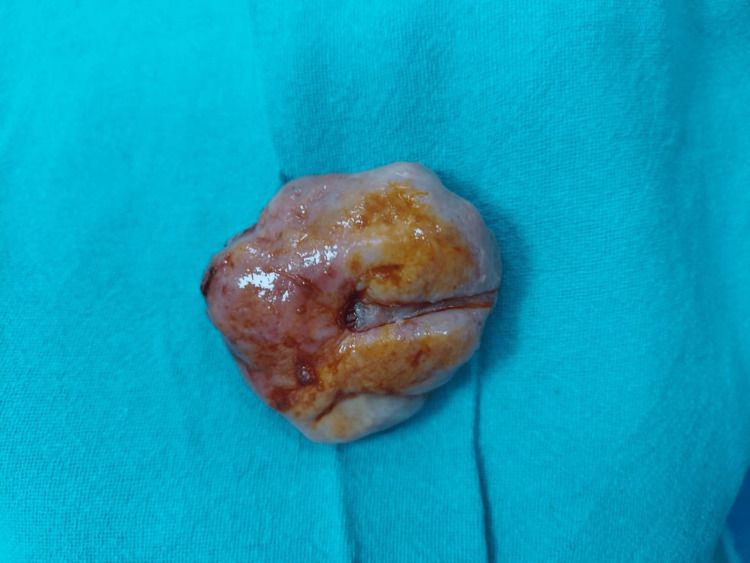
Intramyometrial fibroid

This patient was then taken for surgery after obtaining appropriate consent. Complications such as hemorrhage, massive transfusion, uterine artery embolization (UAE), and emergency hysterectomy were explained. A blood transfusion was started. While examining the operating table (OT) table, the fibroid was found expelled out of the vaginal cavity; hanging onto the pedicle (Figure 4). Diagnosis of spontaneous expulsion of fibroid was made. There was no active bleeding seen after the expulsion of the fibroid. The pedicle which seemed to be arising from the fundus was clamped, cut, and ligated near its origin. Post expulsion, the uterus was normal in size, firm, and freely movable. Uterine packing was done as a precautionary measure. The mass along with the pedicle was sent for histopathological examination which was suggestive of adenomyosis with leiomyoma.

The patient was kept for observation in the surgical intensive care unit (ICU) and one more unit of blood transfusion was given. The uterine pack was removed 24 hours postoperatively. The postoperative period showed no complications, the bleeding had stopped, and her postoperative vitals were within normal limits. The patient was shifted to the ward and was counseled about secondary hemorrhage due to bleeding from the pedicle and the need for UAE or possible hysterectomy only as a necessary emergency intervention. Follow up ultrasound was done with no sign of any uterine mass. The patient was followed up for seven days with an uneventful recovery. She was discharged on iron and vitamin supplements. She was later called on an outdoor basis with no complaints.

## Discussion

Uterine myomas in females are the most common smooth muscle benign solid tumors [1,4]. The incidence of this non-capsulated benign tumor ranges in about 40-50% of females aged 35 years and above [4]. Commonly they are asymptomatic [1] in about 70-80% of cases but in approximately 5% of cases, fibroids can be symptomatic, most often; the submucosal type [5]. The occurrence of uterine fibroids is more often seen in Africans and the Caribbean [2]. The symptomatic leiomyoma may present as menorrhagia, pelvic pain or dysmenorrhoea, infertility, and may cause pregnancy complications. Other associated symptoms may include pressure symptoms, and complication-related symptoms like ulceration, infection, fever, pain, etc. [3]. The hypothesis of leiomyoma is that it may arise from a single neoplastic smooth muscle cell, the stimulus of which arises from a chromosomal abnormality or by stimulation of a growth factor directly or by estrogen. This is also backed by a positive family history [3]. Most of the fibroids which are asymptomatic are often found during the clinical examination or during ultrasonography or other procedures. The symptoms are mostly associated with the site than the size of the fibroid. Hence submucosal type which is the least common type is the most symptomatic [3]. Asymptomatic fibroids do not require any intervention, but symptomatic fibroids can be managed by either medical methods or surgical interventions. Other modalities of treatment include UAE and hysteroscopic resection [4].

Spontaneous expulsion of uterine fibroid is a rare gynecologic event, especially in young females. This study is similar to the study done by Rios [1]. The cause of which is yet unexplained. However, theories suggest it may possibly be due to ischemia followed by tissue necrosis resulting in the expelling of dead tissue. This is most likely seen in menopausal females due to inadequate vascularity. Other causes of spontaneous expulsion may be due to any drug intake, intrauterine device (IUD), cesarean section (CS), or UAE. It may also result from a rupture of the pedicle; courtesy of strong uterine contractions [6,7].

In cases where expulsion has been complete, a thorough examination of the fibroid and the pedicle, if any, should be made. A bleeding pedicle may warrant a pedunculectomy, as in this case. In cases where expulsion is incomplete, muscle constrictors may be used. Coverage of higher antibiotics and postoperative care may suffice in such cases. Post evacuation, monitoring of patients in an ICU facility should be considered. Monitoring of body temperature, vitals, bleeding PV, inflammatory indicators, and Hb levels should be monitored for early diagnosis and prevention of further complications. Requirement for further interventions like UAE or hysterectomy, if need be, can be considered. In cases of partial or incomplete elimination, surgeries like vaginal myomectomy can be considered as it has a good success rate.

## Conclusions

The presence of uterine fibroid among reproductive-age females is very common. Asymptomatic fibroids do not require any intervention. The management in cases of symptomatic fibroids is individualized based on age, fertility, size, and site of myoma and its presenting symptom. Very rarely, as discussed in this case, a pedunculated fibroid may undergo spontaneous expulsion. The expulsion process is usually non-characteristic, however, symptoms can mimic an abortion. Usually, abdominal pain and bleeding PV are most commonly present as in the case here. A prolapsed pedunculated fibroid usually warrants a surgical intervention - either a vaginal or abdominal myomectomy. However, in cases where the self-expulsion of fibroid occurs, the process can be long as the tissue pieces might be excreted in small fragments due to necrosis and decomposition. Such spontaneous expulsion may result in potential morbidity as it can cause diagnostic delay and further delay in management. Furthermore, the necrotic fragments can get infected resulting in pus formation. Sometimes, to aid expulsion surgical evacuation of necrotic fragments may be required. As in this case, a prolapsed pedunculated fibroid was diagnosed intra-operatively and a pediculectomy was warranted.

Thus, a case of complete expulsion of fibroid may sometimes cause significant morbidity and may prolong hospital stay. Clinical suspicion, elimination of pregnancy, and ultrasonography can warrant diagnosis in such cases. A complete and spontaneous expulsion, being an unusual situation, especially in a young female can lead to a delayed diagnosis and can cause significant risk to the patient. Hence, a practitioner should always consider the possibility of myoma degeneration and spontaneous removal in a non-pregnant patient presenting with pain and bleeding PV.

## References

[1] Rios SD, Ribeiro JS, Mota MAS, Chen ACR, Chen JR, de Resende CN (2020). Spontaneous expulsion of a submucosal uterine fibroid without embolization in a pre-menopausal woman. SAGE Open Med Case Rep.

[2] Parker WH (2007). Etiology, symptomatology, and diagnosis of uterine myomas. Fertil Steril.

[3] Buttram VC, Reiter RC (1981). Uterine leiomyomata: etiology, symptomatology, and management. Fertil Steril.

[4] Singh K, Thakur S, Saroha I (2022). Spontaneous expulsion of uterine fibroid vaginally; mimicking inevitable abortion: a case report. Indian J Obstet Gynecol Res.

[5] Marshall LM, Spiegelman D, Barbieri RL (1997). Variation in the incidence of uterine leiomyoma among premenopausal women by age and race. Obstet Gynecol.

[6] Murakami T, Niikura H, Shima Y, Terada Y, Okamura K (2007). Sloughing off of a cervical myoma after cesarean section: a case report. J Reprod Med.

[7] Bagga R, Rai R, Kalra J, Saha PK, Singh T (2017). An unusual cause of postabortal fever requiring prompt surgical intervention: a pyomyoma and its imaging features. Oman Med J.

